# Preoperative neutrophil-to-lymphocyte, platelet-to-lymphocyte and monocyte-to-lymphocyte ratio as a prognostic factor in non-endometrioid endometrial cancer

**DOI:** 10.7150/ijms.64658

**Published:** 2021-09-24

**Authors:** Heekyoung Song, Min Jin Jeong, Jimin Cha, Ji Sun Lee, Ji Geun Yoo, Min Jong Song, Jin Hwi Kim, Sung Jong Lee, Hae Nam Lee, Joo Hee Yoon, Dong Choon Park, Sang Il Kim

**Affiliations:** 1 Department of Obstetrics and Gynecology, Seoul St. Mary's Hospital, College of Medicine, The Catholic University of Korea, Seoul, Republic of Korea.; 2 Department of Obstetrics and Gynecology, Eunpyeong St. Mary's Hospital, College of Medicine, The Catholic University of Korea, Seoul, Republic of Korea.; 3 Department of Obstetrics and Gynecology, Daejeon St. Mary's Hospital, College of Medicine, The Catholic University of Korea, Seoul, Republic of Korea.; 4 Department of Obstetrics and Gynecology, Yeouido St. Mary's hospital, College of Medicine, The Catholic University of Korea, Seoul, Republic of Korea.; 5 Department of Obstetrics and Gynecology, Uijeongbu St. Mary's hospital, College of Medicine, The Catholic University of Korea, Seoul, Republic of Korea.; 6 Department of Obstetrics and Gynecology, Buchen St. Mary's Hospital, College of Medicine, The Catholic University of Korea, Seoul, Republic of Korea.; 7 Department of Obstetrics and Gynecology, St. Vincent's Hospital, College of Medicine, The Catholic University of Korea, Seoul, Republic of Korea.

**Keywords:** endometrial cancer, uterine cancer, high risk, systemic inflammation response, prognostic factors, neutrophil-lymphocyte ratio, platelet-lymphocyte ratio, monocyte-lymphocyte ratio

## Abstract

**Objective:** Evaluate the prognostic value of neutrophil-lymphocyte ratio (NMR), platelet-lymphocyte ratio (PLR), and monocyte-lymphocyte ratio (MLR) in patients with non-endometrioid endometrial cancer.

**Method:** Laboratory and clinicopathological data from 118 patients with non-endometrioid endometrial cancer who underwent surgical resection between January 2010 and December 2019 were reviewed. NLR, PLR and MLR were analyzed for correlations with recurrence and survival. The receiver operating characteristic (ROC) curves were generated for the NLR, PLR, and MLR. Optimal cut-off values were determined as the points at which the Youden index (sensitivity + specificity - 1) was maximal. Based on the results of the ROC curve analysis, the patients were grouped into high MLR and low MLR groups. Recurrence rate, disease-free survival, and overall survival were compared between the two groups. The prognostic factors were investigated using univariate and multivariate Cox proportional hazards model.

**Results:** The optimal cut-off value of MLR was 0.191 (AUC, 0.718; *p* < 0.001). Significantly more patients in the high MLR group experienced recurrence (60.3% vs. 15.6%, *p* < 0.0001) and cancer-related deaths (46.6% vs. 13.3%, *p* = 0.003). In multivariate analysis, advanced stage and high MLR were independent prognostic factors for disease-free survival and overall survival.

**Conclusion:** Elevated MLR was significantly associated poor clinical outcomes in patients with non endometrioid endometrial cancer. Our findings suggest that MLR may be clinically reliable and useful as an independent prognostic marker for patients with non-endometrioid endometrial cancer.

## Introduction

Endometrial cancer (EC) is the fourth most common cancer and the most common gynecological cancer affecting women in developed countries 1. Approximately 66,570 new cases and 12,940 deaths related to EC are expected to occur in the United States in 2021 2. In Korea, the incidence of EC has increased in recent years, and EC is now the most common gynecologic cancer 3.

ECs are divided into two types based on differences in histology and oncologic outcomes 4. Type I, or endometrioid EC, is the most common type and has an excellent prognosis. In contrast, type II, or non-endometrioid EC, which includes serous carcinoma, clear cell carcinoma, carcinosarcoma, and undifferentiated/dedifferentiated carcinoma, has been identified as a distinct, high-risk variant with poor prognosis 5. Unlike endometrioid EC, these subtypes are detected at an advanced stage in approximately 40% of cases, and even in cases where the disease is apparently confined to the uterus, the rate of recurrence is high 6, 7. Despite a multidisciplinary treatment approach involving surgery, radiotherapy, and chemotherapy, a significant number of patients suffer from recurrent disease, and women with advanced-stage or recurrent EC have poor clinical outcomes. Therefore, although these subtypes account for only 10%-20% of all EC cases, they are responsible for approximately 40% of EC-related deaths 8.

Traditional prognostic factors for EC include initial stage, grade, histologic subtype, age at diagnosis, tumor size, and lymphovascular space invasion (LVSI) 9. However, these conventional risk factors are not accurate enough to predict survival outcomes, especially in patients with non-endometrioid EC. As non-endometrioid EC has a high recurrence rate and mortality even in early stage disease, identifying risk factors for recurrence during pre-treatment assessment is crucial to optimize treatment and improve survival.

Recent studies support the role of systemic inflammatory responses in carcinogenesis, progression, and prognosis 10-12. Peripheral blood cells, including neutrophils, lymphocytes, and monocytes, are biomarkers of tumor immunity and play crucial roles in the systemic inflammatory response 13. The neutrophil-lymphocyte ratio (NLR), platelet-lymphocyte ratio (PLR), and monocyte-lymphocyte ratio (MLR) are markers of the systemic inflammatory response 14. NLR, PLR, and MLR have been widely used to predict the prognosis of gynecologic cancer, including EC 15-21. However, most studies to date included patients with endometrioid EC. To the best of our knowledge, the prognostic value of these ratios in patients with non-endometrioid ECs is unclear. Therefore, the aim of this study was to evaluate the prognostic value of NLR, PLR, and MLR in patients with non-endometrioid EC.

## Materials and Methods

This retrospective multicenter study was approved by the Institutional Review Board of the Catholic University of Korea. The requirement for informed consent was waived owing to the nature of the study. The study was conducted in accordance with the principles of the Declaration of Helsinki.

We reviewed our institution's cancer registry and identified patients who underwent primary surgical treatment for non-endometrioid EC from January 2010 to December 2019. All patients who were diagnosed with non-endometrioid EC, including serous carcinoma, clear cell carcinoma, carcinosarcoma, and undifferentiated/dedifferentiated carcinoma, were retrospectively reviewed. Data from 128 patients were recorded in a single database. Patient data were retrieved from five institutions: Seoul St. Mary's Hospital (n = 47), St. Vincent's Hospital (n = 30), Bucheon St. Mary's Hospital (n = 21), Yeouido St. Mary's Hospital (n = 15), and Uijeongbu St. Mary's Hospital (n =15).

We excluded patients who did not undergo primary surgery; those with a history of inflammatory, hematological, or autoimmune diseases; or those who had no preoperative complete blood cell count (CBC) or CBC performed within 1 week before surgery. Patients with incomplete clinicopathological data or follow-up information were excluded. The remaining 118 patients were included in this study.

All patients underwent primary surgical treatment including total hysterectomy, bilateral salpingo-oophorectomy, and systematic lymphadenectomy. And systemic lymphadenectomy includes pelvic and para-aortic lymphadenectomy. Postoperatively, patients were treated with adjuvant chemotherapy, radiation therapy, or a combination of chemotherapy and radiation according to the disease risk factors and at the physician's discretion.

The laboratory tests were individually performed in each hospital. Laboratory results for CBCs included absolute neutrophil count, absolute lymphocyte count, absolute monocyte count, and platelet count. NLR and PLR were defined as the absolute neutrophil count or platelet count divided by the absolute lymphocyte count. Similarly, MLR was defined as the absolute monocyte count divided by the absolute lymphocyte count.

Disease-free survival (DFS) was measured from the date of diagnosis of EC to the date of the first disease progression. If the patient had no recurrence, it was censored at the date of death or at the last follow-up. Overall survival (OS) was measured from the date of initial diagnosis to the date of cancer-related death or the last follow-up. The primary endpoint was the DFS. The secondary endpoint was the OS.

The receiver operating characteristic (ROC) curves of DFS were generated for the NLR, PLR, and MLR. Optimal cut-off values of NLR, PLR, and MLR were determined as the points at which the Youden index (sensitivity + specificity - 1) was maximal. Based on the results of the ROC curve analysis, the patients were grouped into high MLR and low MLR groups.

DFS and OS were analyzed using the Kaplan-Meier method, and curves were compared using the log-rank test. We performed univariate and multivariate analyses using the Cox proportional hazards model to evaluate the effects of the prognostic factors. All statistical analyses were performed using the Statistical Package for the Social Science (SPSS) statistical software package (version 22.0; SPSS Inc., Chicago, IL, USA). Statistical significance was set at P <0.05.

## Results

Overall, 118 patients were included in the final analysis. The baseline characteristics of the patients are presented in Table 1. The median age at diagnosis was 61 years (range, 42-83 years). Sixty-five (55.1%), six (5.1%), 36 (30.5%), and 11 (9.3%) patients had stage I, II, III, and IV disease, respectively. Serous carcinoma was the most common histological subtype (48.3%). In total, 52 (44.1%) patients had LVSI and 37 (31.4%) had lymph node involvement. During a median length of observation of 41 months (range: 3-144 months), 51 (43.2%) patients had tumor recurrence, and 40 (33.9%) died from cancer-related causes.

**Figure 1:**
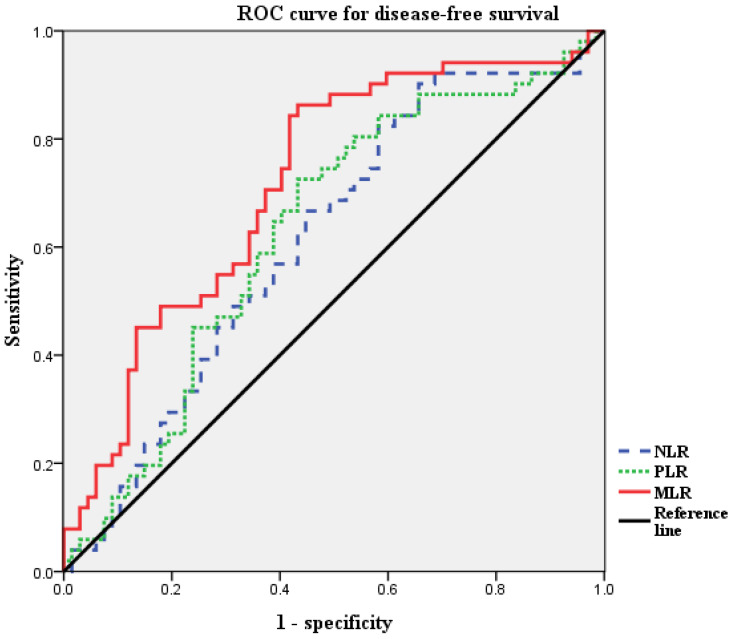
** ROC curves for DFS of NLR, PLR and MLR to predict recurrence.** Optimal NLR, PLR and MLR cut-off value was 1.316, 132.4 and 0.191 respectively. The AUC was 0.615, 0.630 and 0.718. ROC, receiver operating characteristic; AUC, area under the curve; NLR, neutrophil-to-lymphocyte ratio; PLR, platelet-to-lymphocyte ratio; MLR, monocyte-to-lymphocyte ratio.

Next, we defined the thresholds of NLR, PLR, and MLR using ROC curve analysis for our patient population (Figure 1). Median NLR level was 1.95 (range 0.7-18.7). The optimal cut-off value of NLR was 1.316 for DFS (area under the curve [AUC]: 0.615; 95% CI: 0.513-0.717, *p*=0.09). The median PLR level was 139.5 (range, 48.3-434.1). The optimal cut-off value of PLR was 132.4, for DFS (AUC: 0.630; 95% CI: 0.528-0.732, *p*=0.07). The median MLR level was 0.218 (range, 0.065-1.754). The optimal cut-off value of MLR was 0.191 for DFS (AUC, 0.718; 95% CI, 0.624-0.811; *p* < 0.001). According to ROC curve analysis, the AUC of MLR was the highest, and it was the only statistically significant variable. Thus, the MLR cut-off was used to divide the patients into high MLR (MLR ≥0.191) and low MLR (MLR <0.191).

The associations between clinicopathological factors and MLR are shown in Table 2. The low MLR and high MLR groups included 45 (38.1%) and 73 (61.9%) patients, respectively. There were no statistically significant differences between the two groups in terms of age, BMI, and histologic subtype. Significant differences between the two groups were demonstrated for the following categorical variables: FIGO stage (*p* = 0.001), LVSI (*p* = 0.004), and LN metastasis (*p* = 0.003). Interestingly, significantly more patients in the high MLR group experienced recurrence (60.3% vs. 15.6%, *p* < 0.0001) and cancer-related deaths (46.6% vs. 13.3%, *p* = 0.003).

Cox's proportional hazards model was used to evaluate the prognostic factors for recurrence (Table 3). Univariate analysis revealed that DFS was significantly associated with factors other than age. However, in multivariate analysis, only advanced stage (adjusted HR: 2.473; 95% CI: 1.389-4.405; *p* = 0.002) and high MLR (adjusted HR: 3.647; 95% CI: 1.60-8.315; *p* = 0.002) were independent prognostic factors for DFS.

Univariate analysis revealed that OS was significantly associated with advanced stage, LVSI, LN metastasis, high NLR, high PLR, and high MLR (Table 4). However, in multivariate analysis, only advanced stage (adjusted HR: 2.930; 95% CI: 1.210-5.767; *p* =0.002) and high MLR (adjusted HR: 2.941; 95% CI: 1.21-7.147; *p* = 0.017) retained their prognostic significance for OS.

**Figure 2:**
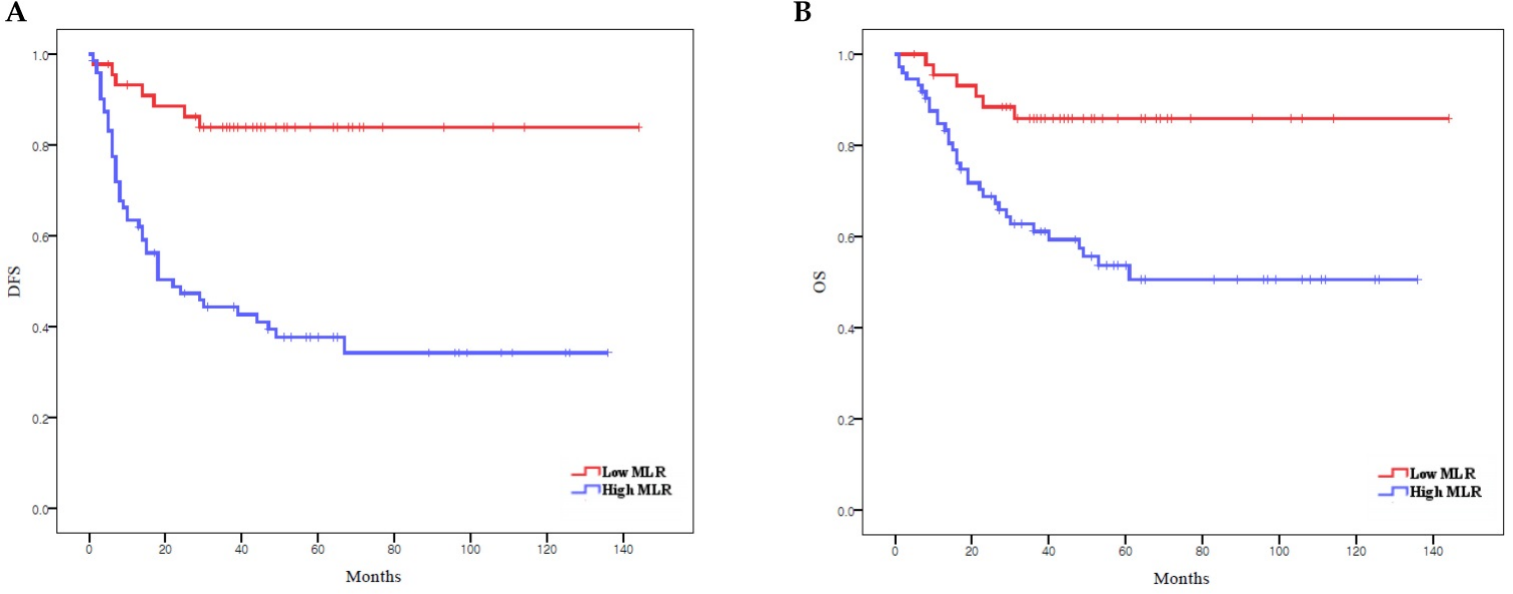
Survival curves according to MLR: (**A**) Kaplan-Meier survival curves for DFS of patients with a high MLR and those with a low MLR. (**B**) Kaplan-Meier survival curves for OS of patients with a high MLR and those with a low MLR. DFS, disease-free survival; OS, overall survival; MLR, monocyte-to-lymphocyte ratio.

According to Kaplan-Meier analysis, the 5-year DFS rates in the low and high MLR groups were 83.8% and 37.7% (*p* < 0.0001), respectively, and the 5-year OS rates in these two groups were 85.9% and 51.0%, respectively (*p* = 0.001) (Figure 2). Both DFS and OS were significantly lower in the high MLR group than in the low MLR group.

## Discussion

The findings of this study indicate that the preoperative MLR ratio is an independent predictor of recurrence and survival in patients with non-endometrioid EC. Compared to endometrioid ECs, non-endometrioid ECs have poorer survival outcomes 5-8. Thus, it is necessary to identify novel risk factors for recurrence. To our knowledge, this is the first study to describe an association between pre-treatment MLR and prognosis in non-endometrioid ECs. Our findings provide valuable insight into the predictive potential of the systemic inflammatory response on the oncologic outcomes of non-endometrioid EC, including recurrence and cancer-related deaths.

In 1863, Virchow first described the association between inflammation and cancer 22. Since then, numerous studies have suggested that inflammatory cells and cytokines are likely to contribute to tumor growth, progression, and metastasis 23, 24. Previous studies have reported an association between the systemic inflammatory response and prognosis of gynecologic cancer, including EC 15-21. However, in most existing studies on EC, the majority of patients had endometrioid EC. Haruma et al. reported pre-treatment NLR as a predictor of poor prognosis in EC. Among the 320 patients included in the study, only 46 had non-endometrioid histology 25. Another study by Eo et al. reported the lymphocyte-to-monocyte ratio (LMR) as an independent prognostic factor for both DFS and OS after surgical resection in patients with EC. However, only 21 of 255 patients had non-endometrioid histology 26.

Non-endometrioid EC is relatively rare but is more aggressive and has more distant metastasis at diagnosis, and a worse survival rate, than endometrioid EC. Therefore, adjuvant chemotherapy with or without vaginal brachytherapy is preferred, even in women with early stage disease 27. Given the association between systemic inflammation and cancer progression and metastasis, we hypothesized that the systemic inflammatory response of non-endometrioid EC differs from endometrioid EC. Thus, we sought to identify novel prognostic indicators of non-endometrioid EC. In the present study, the prognostic impact of the NLR and PLR on DFS and OS was demonstrated by univariate analysis, but the significance of the associations was lost on multivariate analysis. Only MLR was demonstrated as a surrogate marker for both DFS and OS on multivariate analysis. These results are in concordance with a previous study, which suggested that MLR is associated with survival in patients with colorectal cancer, ovarian cancer, and hepatocellular carcinoma 28-30.

The mechanisms underlying the association between high MLR and poor outcomes remain poorly understood. Various studies have reported the pro-tumoral functions of monocytes, such as differentiation into tumor-associated macrophages (TAMs), metastatic cell seeding, suppression of T cell function, angiogenesis, and extracellular matrix remodeling 31. TAMs produce various factors, such as tumors. Growth factors and angiogenic factors accelerate tumor progression and invasion 32. Monocytes can also promote immune escape by limiting activated CD8 + T-cell infiltration into the tumor microenvironment 33. Lymphocytes, in contrast, are known for their anti-tumor functions. CD8+ T lymphocytes play a vital role in the cytotoxicity of tumor cells. CD4+ T lymphocytes elicit a vigorous anti-tumor immune response 34. Thus, a high monocyte count and low lymphocyte count may be associated with poor prognosis. Elevated MLR is due to a relative increase in monocyte count or a relative decrease in lymphocyte count. The imbalance between the unfavorable role of monocytes and the favorable role of lymphocytes in the tumor microenvironment plays a role in cancer progression. Thus, MLR might be an inflammatory marker reflecting increased cancer aggressiveness.

Our results indicate that MLR is associated with survival in patients with non-endometrioid EC, which suggest that the immune system is important in this disease. It might be possible to identify patients who are at high risk of recurrence or death after the standard treatment. Therefore, MLR can be used to change the treatment strategy even in the early stage cases. Also, we can consider further aggressive treatment in patients with high MLR.

Our study has several limitations. First, this was a retrospective study. Second, the sample size may have been insufficient. However, unlike other studies that included both endometrioid and non-endometrioid EC patients, we included only non-endometrioid ECs and the number of patients with non-endometrioid EC was comparable to that in other retrospective studies. Third, as there was no defined MLR value for non-endometrioid EC patients, we had to set a cut-off value for our study. Nevertheless, our study is valuable as we found that an elevated MLR was significantly associated with DFS and OS in patients with non-endometrioid EC.

In conclusion, our findings suggest that MLR may be clinically reliable and useful as an independent prognostic marker for patients with non-endometrioid EC. Further studies are needed to confirm our findings and to determine appropriate cut-off values.

## Figures and Tables

**Table 1:** Baseline patient characteristics (n = 118)

	No. of patients	%
Age (years), median (range)	61	42-83
BMI (kg/m^2^), median (range)	24.87	16.67-34.39
**FIGO stage**		
I	65	55.1
II	6	5.1
III	36	30.5
IV	11	9.3
**Histology**		
Serous	57	48.3
Clear	22	18.7
Carcinosarcoma	30	25.4
Un/Dedifferentiated	9	7.6
**LVSI**		
Absent	66	55.9
Positive	52	44.1
**LN metastasis**		
Absent	81	68.6
Positive	37	31.4
Median follow-up (months)	41	
Range	3-144	
Overall recurrences	51	43.2
Deaths	40	33.9

BMI, body mass index; FIGO, International Federation of Gynecology and Obstetrics; LVSI, lymphovascular space invasion; LN, lymph node.

**Table 2:** Clinical and pathological characteristics according to the MLR (n=118)

	Low MLR group (n = 45, %)	High MLR group (n = 73, %)	*p* value
Age (years), median (range)	61 (52-77)	62 (42-83)	0.932
BMI (kg/m^2^), median (range)	24.9 (16.7-31.6)	24.7 (18.0-34.4)	0.392
**FIGO stage**			0.001
I	33 (73.3)	32 (43.8)	
II	3 (6.7)	3 (4.1)	
III	7 (15.6)	29 (39.7)	
IV	2 (4.4)	9 (12.4)	
**Histology**			0.734
Serous	20 (44.4)	37 (50.7)	
Clear	11 (24.5)	11 (15.1)	
Carcinosarcoma	10 (22.2)	20 (27.4)	
Un/Dedifferentiated	4 (8.9)	5 (6.8)	
**LVSI**			0.004
Absent	33 (73.3)	33 (45.2)	
Positive	12 (26.7)	40 (54.8)	
**LN metastasis**			0.003
Absent	38 (84.4)	43 (58.9)	
Positive	7 (15.6)	30 (41.1)	
Recurrence	7 (15.6)	44 (60.3)	< 0.0001
Death	6 (13.3)	34 (46.6)	0.003

MLR, monocyte-to-lymphocyte ratio; BMI, body mass index; FIGO, International Federation of Gynecology and Obstetrics; LVSI, lymphovascular space invasion; LN, lymph node.

**Table 3:** Univariate and multivariate analysis of prognostic factors for disease-free survival (n = 118)

Characteristics	Univariate analysis			Multivariate analysis		
	OR	95% CI	*p value*	OR	95% CI	*p value*
**Age, years**						
<60	1 (Ref)	-	-			
≥60	1.392	0.762-2.543	0.282			
**FIGO stage**						
I	1 (Ref)	-	-	1 (Ref)	-	-
II-IV	2.976	1.682-5.268	< 0.001^*^	2.420	1.357 - 4.319	0.003^*^
**LVSI**						
No	1 (Ref)	-	-	1 (Ref)	-	-
Yes	2.345	1.344-4.091	0.003^*^	1.826	0.773 - 4.963	0.171
**LN metastasis**						
No	1 (Ref)	-	-	1 (Ref)	-	-
Yes	2.717	1.563-4.723	< 0.001^*^	1.030	0.419 - 2.534	0.948
**NLR**						
< 1.316	1 (Ref)	-	-	1 (Ref)	-	-
≥ 1.316	3.670	1.457-9.247	0.006^*^	1.797	0.680 - 4.751	0.237
**PLR**						
< 132.4	1 (Ref)	-	-	1 (Ref)	-	-
≥ 132.4	2.408	1.354-4.283	0.003^*^	1.555	0.853 - 2.837	0.150
**MLR**						
< 0.191	1 (Ref)	-	-	1 (Ref)	-	-
≥ 0.191	5.245	2.359-11.665	< 0.001^*^	3.647	1.600 - 8.315	0.002^*^

Covariates with *p* < 0.05 on univariate analysis were included in multivariate model.OR, odds ratio; CI, confidence interval; Ref, reference; FIGO, International Federation of Gynecology and Obstetrics; LVSI, lymphovascular space invasion; LN, lymph node; NLR, neutrophil-to-lymphocyte ratio; PLR, platelet-to-lymphocyte ratio; MLR, monocyte-to-lymphocyte ratio.

**Table 4:** Univariate and multivariate analysis of prognostic factors for overall survival (n = 118)

Characteristics	Univariate analysis			Multivariate analysis		
	OR	95% CI	*p value*	OR	95% CI	*p value*
**Age, years**						
<60	1 (Ref)	-	-			
≥60	1.314	0.663-2.604	0.434			
**FIGO stage**						
I	1 (Ref)	-	-	1 (Ref)	-	-
II-IV	3.616	1.820-7.186	< 0.001^*^	2.980	1.487-5.974	0.002^*^
**LVSI**						
No	1 (Ref)	-	-	1 (Ref)	-	-
Yes	2.935	1.514-5.687	0.001^*^	1.413	0.562-3.553	0.462
**LN metastasis**						
No	1 (Ref)	-	-	1 (Ref)	-	-
Yes	2.786	1.472-5.273	0.002^*^	0.822	0.294-2.297	0.708
NLR						
< 1.316	1 (Ref)	-	-	1 (Ref)	-	-
≥ 1.316	3.048	1.081-8.594	0.035^*^	1.365	0.454-4.102	0.579
**PLR**						
< 132.4	1 (Ref)	-	-	1 (Ref)	-	-
≥ 132.4	2.427	1.224-4.812	0.011^*^	1.590	0.775-3.260	0.206
**MLR**						
< 0.191	1 (Ref)	-	-	1 (Ref)	-	-
≥ 0.191	3.854	1.610-9.225	0.001^*^	2.941	1.210-7.147	0.017^*^

Covariates with *p* < 0.05 on univariate analysis were included in multivariate model.OR, odds ratio; CI, confidence interval; Ref, reference; FIGO, International Federation of Gynecology and Obstetrics; LVSI, lymphovascular space invasion; LN, lymph node; NLR, neutrophil-to-lymphocyte ratio; PLR, platelet-to-lymphocyte ratio; MLR, monocyte-to-lymphocyte ratio.
